# Stochastic Identification of Guided Wave Propagation under Ambient Temperature via Non-Stationary Time Series Models

**DOI:** 10.3390/s21165672

**Published:** 2021-08-23

**Authors:** Shabbir Ahmed, Fotis Kopsaftopoulos

**Affiliations:** Intelligent Structural Systems Laboratory (ISSL), Mechanical, Aerospace and Nuclear Engineering, Rensselaer Polytechnic Institute, Troy, NY 12180, USA; ahmeds6@rpi.edu

**Keywords:** guided waves, stochastic identification, time-varying models, piezoelectric sensors, time series models, non-stationary models

## Abstract

In the context of active-sensing guided-wave-based acousto-ultrasound structural health monitoring, environmental and operational variability poses a considerable challenge in the damage diagnosis process as they may mask the presence of damage. In this work, the stochastic nature of guided wave propagation due to the small temperature variation, naturally occurring in the ambient or environment, is rigorously investigated and modeled with the help of stochastic time-varying time series models, for the first time, with a system identification point of view. More specifically, the output-only recursive maximum likelihood time-varying auto-regressive model (RML-TAR) is employed to investigate the uncertainty in guided wave propagation by analyzing the time-varying model parameters. The steps and facets of the identification procedure are presented, and the obtained model is used for modeling the uncertainty of the time-varying model parameters that capture the underlying dynamics of the guided waves. The stochasticity inherent in the modal properties of the system, such as natural frequencies and damping ratios, is also analyzed with the help of the identified RML-TAR model. It is stressed that the narrow-band high-frequency actuation for guided wave propagation excites more than one frequency in the system. The values and the time evolution of those frequencies are analyzed, and the associated uncertainties are also investigated. In addition, a high-fidelity finite element (FE) model was established and Monte Carlo simulations on that FE model were carried out to understand the effect of small temperature perturbation on guided wave signals.

**Important conventions and symbols**

Definition is indicated by :=. Matrix transposition is indicated by the superscript *T*.

Bold-face upper/lower case symbols designate matrix/column vector quantities, respectively.

A functional argument in parentheses designates the function of a real variable; for instance P(x) is a function of the real variable *x*.

A functional argument in brackets designates the function of an integer variable; for instance x[t] is a function of normalized discrete time (t=1,2,…). The conversion from discrete normalized time to analog time is based on $\left(t-1\right)T_{s}$, with $T_{s}$ designating the sampling period.

A hat designates estimator/estimate; for instance $\hat{\theta }$ is an estimator/estimate of θ.

## 1. Introduction

Structural health monitoring (SHM) refers to the process of damage detection, localization, and quantification within a structure, which may be collectively referred to as damage diagnosis [1,2]. The recent emphasis on increased safety, enhanced reliability, robust and reliable self-sensing, and self-diagnostic capabilities have made it indispensable to incorporate SHM systems into aircraft, civil, and mechanical structures [3]. The main classification of the methods used in SHM can be local (for a small inspection area where damage is suspected, also referred to as a “hotspot”) or global (monitoring the complete structure and its dynamic response) based on the nature of the interrogating region of the structure. In addition, SHM methods can also be classified as active or passive based on the usage of active actuation/excitation signals to actively interrogate the structure.

**Figure 1:**
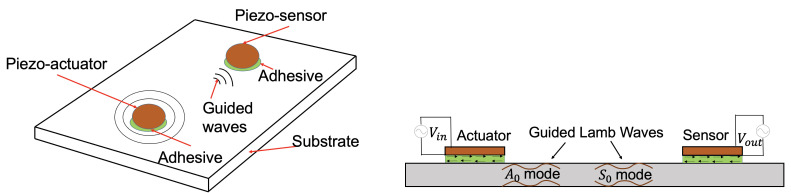
Schematic diagram of the generation and sensing of guided wave propagation in a thin plate via two PZT transducers acting as actuators and sensors.

One of the most prominent active-sensing, local SHM family of methods is based on the use of ultrasonic guided waves, i.e., elastic stress waves generated at ultrasonic frequencies that propagate on thin structures while also being reflected on the surface boundaries [4] (Chapter 5, pp. 311–315), [5] (Chapter 8, pp. 432–446). As these waves propagate through the reflection of surface boundaries, they can travel long distances without significant energy loss. These waves exist in two basic types in thin plates: symmetric and anti-symmetric [6] (Chapter 6, pp. 79–86). Theoretically speaking, for each propagation type, within a thin infinite plate, there exists an infinite number of propagation modes. That is, a finite body can support an infinite number of different guided wave modes. The symmetric modes are designated as $S_{0}$, $S_{1}$, $S_{2}$, *…*, whereas anti-symmetric modes are designated as $A_{0}$, $A_{1}$, $A_{2}$, *…*, and so on [6] (Chapter 6, p. 89), [7] (Chapter 2, p. 119). The governing equations of ultrasonic wave propagation can be solved via various numerical approaches [6,7]. At lower frequencies, the symmetric and anti-symmetric guided waves approach the behavior of the axial plate waves and flexural waves, respectively. Guided waves are highly dispersive in nature and usually the $S_{0}$ and $A_{0}$ modes are considered for the damage diagnosis process because these wave packets sustain the least amount of dispersion [8] (Chapter 6, p. 295, 306). It is also easy to generate and detect these wave packets as they exist during the entire frequency spectrum of the dispersion curve. Figure 1 shows a pictorial representation of the generation and sensing of guided wave propagation in a thin plate. Guided waves can be considered as time-varying signals; therefore, proper modeling techniques are necessary for their accurate representation.

In the context of active-sensing guided-wave-based SHM [1,9,10], the concept of a damage/health index/indicator (D/HI) [11,12] is widely used for tackling damage detection; the obtained features are usually based on the $S_{0}$ and $A_{0}$ modes of the wave propagation signals, such as the time of flight (ToF), the signal amplitude, the energy content, etc. and are used to differentiate between a healthy and a damaged structure [13,14]. The basic idea is that the presence of damage results in a scatter signal when subtracted from a baseline or reference signal. Such DI-based approaches have been used widely in the literature for their inherent simplicity and effectiveness in damage detection in controlled environments; however, these methods are deterministic in nature, and in the face of stochastic, inherently time-varying, and potentially non-linear structural responses, their applicability may become limited [15,16,17]. Under the presence of varying environmental and operating conditions (EOC), traditional DI-based damage detection schemes face significant challenges as the EOC-induced signal changes and corresponding scatter may be on the same order of magnitude, or even exceed, that induced by structural damage itself [18,19]. As a result, the presence of varying EOC may mask the effects of and/or provide a false indication of the presence of damage [20], thus accurate and robust stochastic modeling techniques are needed to represent the wave propagation in the face of uncertainty.

Among the effects of different EOCs on ultrasonic wave propagation, the presence of temperature is a prominent one that has been addressed in the literature [21,22,23,24,25,26,27], as it results in changes in the elastic properties of the materials (host structural, adhesive, and piezoelectric), which in turn, affect the propagating wave characteristics. It has also been reported that changes in temperature result in non-linear changes in the wave propagation properties [18]. Several data-based modeling techniques have been proposed in a number of studies where a deterministic linear relationship was assumed between the temperature and the time of flight as well as the amplitude of the $S_{0}$ and $A_{0}$ modes. This type of deterministic modeling constitutes the basis for a range of temperature compensation techniques. The baseline modification methods such as optimal baseline selection (OBS) [28,29], baseline signal stretch (BSS) [30], and their variants [31,32,33], where the baseline signal is selected, modified, or stretched in such a way so that the effect of temperature change is minimized in the scatter signal.

Under the physics-based modeling family of methods, both analytical and numerical modeling approaches have been proposed. Analytical modeling of wave propagation is limited to simple geometries such as thin plates without accounting for boundaries and side reflections, and elastic half-space, i.e., accounting for reflections only from the top surface [4,5,8,34]. In order to model guided wave propagation in complex geometries, the finite element and spectral element methods (F/SEM) have been widely used; however, it is extremely challenging to accurately model higher than the first two modes, i.e., $S_{0}$ and $A_{0}$, along with the effects of boundary reflections, especially when it comes to composite materials [35,36]. In addition, temperature-induced signal non-linearities arise due to the “shear lag” effect and the presence of the adhesive layer on the plate–PZT interface [37,38]. Ha et al. [39] have modeled the effect of temperature on the adhesive layer for guided wave propagation through numerical simulations using SEM. The investigation and compensation of increasing temperature on the material properties and the subsequent effects on the wave propagation were addressed by Roy et al. [18] using a physics-based method leveraging SEM and requiring the collection of a set of experimental data sets.

However, in principle F/SEM-based methods are deterministic and computationally expensive, especially when it comes to large complex structures [40,41,42], thus they do not inherently account for the stochastic nature of guided wave propagation. Furthermore, the forward uncertainty quantification can be realized with a significant computational cost and potentially limited accuracy. In a recent study by the authors, it has been reported that guided wave signals generated by piezoelectric transducers mounted on a thin aluminum plate via the use of adhesive can exhibit a significant variation even in a controlled lab environment under minor ambient temperature variations or other unobservable/unknown sources [41]. That is, as expected, there is a presence of stochasticity in guided wave propagation that necessitates the use of appropriate modeling techniques. To the authors’ best knowledge, no such studies, either physics- or data-based, have been reported in the current literature, regarding the modeling of guided wave propagation under uncertainty under ambient conditions. The need for proper modeling of guided wave propagation in the face of uncertainty is of paramount importance in order to enable the development of accurate and reliable damage diagnosis methods under varying EOC. The aspects that still need to be considered may be summarized as follows:

- Investigation of the stochastic and non-stationary nature of guided wave propagation under ambient temperature variation;
- Mathematical modeling of the uncertainty in guided wave propagation due to ambient temperature variation;
- Investigation on the time-varying modal characteristics (natural frequencies and damping ratios) under the influence of ambient temperature as the guided wave propagates through the structure.

In order to tackle these challenges, system identification techniques employing stochastic time-varying (non-stationary) time series models appear as a promising approach to study the stochastic and non-stationary nature of guided wave propagation. In the context of the vibration-based damage diagnosis process, principles of system identification have been widely used to detect structural damage via both stationary and non-stationary time series models [16,43,44,45,46]. Recently, eigen perturbation, Kalman filter, and time-varying auto-regressive (TAR) model-based methods have been used for real-time damage detection in the context of vibration-based damage diagnosis [47,48]. These methods are data-based rather than physics-based and fundamentally of the inverse type. Being statistical in nature, they may offer the advantage of assimilating inherent uncertainty and obviate the need for formulating detailed physics-based F/SEM models. Unlike physics-based methods, they are capable of capturing different uncertainties without having recourse to subjectively assuming the uncertainty in structural parameters. These methods may be broadly classified as non-parametric or parametric.

Non-parametric model structures are characterized by the property that the resulting models are curves or functions that do not explicitly employ a finite-dimensional parameter vector. Non-parametric methods include time-domain models such as the auto-correlation (ACF) and cross-correlation (CCF) [46,48,49,50] functions as well as frequency-domain models, such as the power spectral density (PSD) and the frequency response function (FRF) [46,48,51]. The above mentioned non-parametric methods provide information either on the time or frequency domain. They do not provide any information on how time and frequency changes simultaneously. Time–frequency analysis provides a suitable means to analyze non-stationary signals in the time and frequency domains simultaneously. Such methods include the widely used spectrogram based on the short-time Fourier transform (STFT) [52] and its ramifications, distributions such as the Wigner–Ville and Choi–Williams that are unified under the Cohen class of distributions [53]. In addition, methods based on continuous wavelet transform (CWT) and cross-wavelet transform (XWT) can also be used to extract time–frequency information [54]. Recently, least-squares wavelet analysis (LSWA) and least-squares cross-wavelet analysis (LSCWA) have also been proposed; these methods do not rely on any pre-processing of the measurements and can provide accurate instantaneous frequency information along with phase differences in the time–frequency domain. The XWT and LSCWA cross-spectrograms can potentially show the coherency between two signals in the time–frequency domain and reveal the coupling of the time series components (lead/lag) within each time–frequency neighborhood [55,56].

On the other hand, parametric model structures are parameterized in terms of a parameter vector to be estimated from the available signals. Parametric model structures can be time-invariant as well as time-varying. Time-varying parametric methods are based upon parameterized representations of the time dependent auto-regressive moving average (TARMA) or related types and their extensions. These representations differ from their conventional, stationary, counterparts in that their parameters are time-dependent [16,48,57,58,59]. The methods based upon them are known to offer a number of potential advantages [58,60,61] such as representation parsimony, as models may be potentially specified by a limited number of parameters, improved accuracy, resolution, and tracking of the time-varying dynamics, flexibility in analysis, as parametric methods are capable of directly capturing the underlying structural dynamics responsible for the non-stationary behavior, and flexibility in synthesis (simulation) and prediction. Parametric time-varying methods may be further classified according to the type of structure imposed upon the evolution of the time-varying model parameters, namely: (i) the class of unstructured parameter evolution methods [48,62,63], which impose no particular structure on the evolution of time-varying model parameters; (ii) the class of stochastic parameter evolution methods [48,64,65], which impose stochastic structure on the evolution of the time-varying parameters; (iii) the class of deterministic parameter evolution methods [16,48,66,67], which impose deterministic structure upon the evolution of time-varying parameters. From the above discussion on the literature of temperature compensation of guided waves for damage detection and a brief overview of time-varying models, it is clear that those models have not been used in the context of guided wave-based modeling and SHM, although they seem to offer a number of important characteristics.

In this work, a novel stochastic modeling and analysis framework is proposed for the effective representation of non-stationary guided wave propagation signals via the use of time-varying auto-regressive models (TAR) under ambient temperature variation. A series of laboratory experiments on an aluminum plate outfitted with a network of piezoelectric sensors was performed during eleven days so that the effects of ambient temperature variation could be reflected in the recorded signals. In addition, high-fidelity FEM simulations were performed to investigate the variation of the wave propagation signals under the assumption of normally distributed temperature. Initially, the uncertainty quantification of the obtained signals was performed; next, the stochastic identification via time-varying AR models was performed via a recursive maximum likelihood (RML) estimation scheme along with the investigation of the parameter estimation and modal uncertainty. The main contributions of this study are:

(a) Investigation of the stochastic nature of guided wave propagation in an aluminum plate under ambient temperature variation by means of experimental investigation and high-fidelity physics-based finite element modeling.
(b) Introduction of a data-based stochastic time-varying identification framework via RML parameter estimation for tracking the dynamics of non-stationary guided wave signals.
(c) Investigation of the model-based and experimental modeling and quantification of uncertainty in guided wave propagation due to ambient temperature variation with the help of the RML-TAR time series models.
(d) Extraction and assessment of the RML-based time-dependent modal properties, i.e., natural frequencies and damping ratios of the system under the influence of ambient temperature variation as guided waves propagate on the structure.

The remainder of the paper is organized as follows: a concise overview of non-stationary time series signal representations is presented in Section 2. The theory and steps of the identification procedure are discussed in Section 3. The process of data generation with the help of experimental set-up and the FEM is discussed in Section 4 and the model-based analysis of guided wave propagation under ambient temperature variation is discussed in Section 5. Finally, concluding remarks are summarized in Section 6.

## 2. Non-Stationary Guided Wave Signal Representation

The proper parametric representation of time-varying wave propagation requires the use of appropriate non-stationary stochastic model structures. For a systematic review of both non-parametric and parametric non-stationary models the reader is referred to [48]. Typical models are of the TARMA, i.e., time-varying autoregressive moving average, type or proper extensions (for instance TARMAX representations—that is TARMA representations with eXogenous excitation, which additionally account for measurable excitation [48]). In this study, the parametric time-varying autoregressive (TAR) model structure has been used to represent guided wave propagation signals due to its simple but effective form and parameter estimation methods. TAR models are parameterized representations based on their conventional, stationary AR counterparts with the significant difference being that they allow their parameters to depend upon time. A time-varying auto-regressive TAR(na) model, with na designating its autoregressive order, is thus of the form:

(1) $$y\left[t\right]+\sum_{i=1}^{na}a_{i}\left[t\right]\cdot y\left[t-i\right]=e\left[t\right]\;e\left[t\right]∼\;\mathrm{iid}\;N\left(0,\sigma_{e}^{2}\left[t\right]\right)$$

with *t* designating its discrete time, y[t] the time-varying signal to be modeled, e[t] an (unobservable) uncorrelated (white) innovations sequence with zero mean and time-dependent variance $\sigma_{e}^{2}\left[t\right]$ that generates y[t], and $a_{i}\left[t\right]$, the model’s time-dependent AR parameters. N(·,·) stands for normal distribution with the indicated mean and variance [48,62,68].

It can be shown that the minimum mean square error (MMSE) one-step-ahead prediction $\hat{y}\left[t/t-1\right]$ of the signal value y[t] made at time t−1 (that is for given values of the signal up to time t−1) is (A hat designates estimator/estimate; for instance $\hat{\theta }$ is an estimator/estimate of θ):

(2) $$\hat{y}\left[t/t-1\right]=-\sum_{i=1}^{na}a_{i}\left[t\right]\cdot y\left[t-i\right]$$

Comparing this with the TAR model of Equation (1), it is evident that the one-step-ahead prediction error is equal to e[t], that is:

(3) $$\hat{e}\left[t/t-1\right]≜y\left[t\right]-\hat{y}\left[t/t-1\right]=e\left[t\right]$$

This is an important observation, as it indicates that, just as in the stationary case, the model’s one-step-ahead prediction error (also referred to as the residual) coincides with the (uncorrelated) innovations generating the signal. This is valid as long as the true model parameters of Equation (1) are used in the predictor Equation (2).

Using the backshift operator $B(B^{i}\cdot y\left[t\right]≜y\left[t-i\right])$, the TAR representation of Equation (1) may be compactly re-written as:

(4) $$y\left[t\right]+\sum_{i=1}^{na}a_{i}\left[t\right]\cdot B^{i}\cdot y\left[t\right]=e\left[t\right]\;\Leftrightarrow \;A\left[B,t\right]\cdot y\left[t\right]=e\left[t\right],\;e\left[t\right]∼\;\mathrm{iid}\;N\left(0,\sigma_{e}^{2}\left[t\right]\right)$$

with

(5) $$A\left[B,t\right]=1+\sum_{i=1}^{na}a_{i}\left[t\right]\cdot B^{i}$$

The polynomial A[B,t] is referred to as the AR time-dependent polynomial operator. Note that one may also define $a_{0}\left[t\right]=1$.

## 3. Non-Stationary TAR Model Identification

Given a single, *N*-sample long, non-stationary, guided wave propagation signal record (realization) $y^{N}≜\left\{y\left[1\right]\;y\left[2\right]\;\ldots \;y\left[N\right]\right\}$ the TAR identification problem may be stated as the problem of selecting the corresponding model “structure”, the model AR parameters $a_{i}\left[t\right]$ and the innovations variance $\sigma_{e}^{2}\left[t\right]$ that “best” fit the available measurements. Model “fitness” may be understood in various ways, a common approach being in terms of the model’s predictive ability. This implies that the “best” model is the one characterized by minimal one-step-ahead prediction error. The methods based upon this principle, minimize a function of the prediction error sequence (typically the residual sum of squares (RSS)) and are referred to as the prediction error methods (PEM) [48].

In the case of TAR models, the model structure selection consists of determining the AR order na. More formally, the identification problem may be defined as the selection of the best fitting model from the set G of TAR models corresponding to a particular class:

(6) $$G\;≜\;\left\{M\left(\theta^{N},{\left(\sigma_{e}^{2}\right)}^{N}\right)\;:\;y\left[t\right]+\sum_{i=1}^{na}a_{i}\left[t,\theta \left[t\right]\right]\cdot y\left[t-i\right]=e\left[t,\theta^{t}\right]\right\}$$

and

$$\sigma_{e}^{2}\left[t,\theta^{t}\right]=E\left\{e^{2}\left[t,\theta^{t}\right]\right\},\;t=1,\ldots ,N$$

In this expression, E{·} designates statistical expectation and θ[t] designates the instantaneous AR parameter vector at *t* time instant (Bold-face upper/lower case symbols designate matrix/column vector quantities, respectively; matrix transposition is designated by the superscript *T*):

(7) $$\theta \left[t\right]={\left[a_{1}\left[t\right]\;a_{2}\left[t\right]\;\ldots \;a_{na}\left[t\right]\right]}_{\left[na\times 1\right]}^{T}$$

While $\theta^{t}$ stands for all AR parameters up to time *t*, that is,

(8) $$\theta^{t}≜{\left[\theta^{T}\left[1\right]\;\theta^{T}\left[2\right]\;\theta^{T}\left[3\right]\;\ldots \;\theta^{T}\left[t\right]\right]}_{\left[nat\times 1\right]}^{T}$$

Moreover, $\theta^{N}$ designates all AR parameters at all time instants, and, similarly, ${\left(\sigma_{e}^{2}\right)}^{N}$ the residual variance at all time instants:

(9) $${\left(\sigma_{e}^{2}\right)}^{N}≜\left\{\sigma_{e}^{2}\left[1\right],\;\sigma_{e}^{2}\left[2\right],\;\sigma_{e}^{2}\left[3\right],\;\ldots ,\sigma_{e}^{2}\left[N\right]\right\}$$

The TAR model to be estimated is explicitly parameterized in terms of the specific parameters to be estimated. Thus, both the AR parameters and the one-step-ahead prediction error (residual) signal are designated as functions of these parameters. For the residual signal, in particular, this signifies the fact that it is obtained based upon the current model parameters and the available guided wave signal $y^{N}$ (using the TAR model expression in Equation (6)). Thus, the “best” model may be selected/estimated as the model with parameters that minimize the prediction error.

The model identification problem is usually distinguished into two subproblems: (i) the *parameter estimation* subproblem, and (ii) the *model structure selection* subproblem, presented in Section 3.1 and Section 3.2, respectively.

### 3.1. Model Parameter Estimation

Model parameter estimation refers to the determination, for a given model structure, of the AR parameter vector θ[t] and the residual variance $\sigma_{e}^{2}\left[t\right]$. For a review of the main estimation methods for non-stationary stochastic time series models please see [48]. In the case of TAR models, recursive or adaptive estimation methods provide an accurate and robust parameter estimate. TAR/TARMA models estimated recursively are also referred to as RAR/RARMA models.

Although there are several variations of recursive methods, in the present work we employ a method based on the following exponentially weighted prediction error criterion [48,68]:

(10) $$\hat{\theta }\left[t\right]=\arg\min_{\theta [t]}\sum_{\tau =1}^{t}\lambda^{t-\tau }\cdot e^{2}\left[\tau ,\theta^{\tau -1}\right],$$

with

(11) $$e\left[t,\theta^{t-1}\right]≜y\left[t\right]-\sum_{i=1}^{na}a_{i}\left[t-1\right]\cdot y\left[t-i\right]\approx e\left[t,\theta^{t}\right]$$

In these expressions argmin designates minimizing argument, and $e[t,\theta^{t-1}]$ the model’s one-step-ahead prediction error made at time t−1 without knowing the model parameter values at time *t* as it would be normally necessary. Of course, as indicated in the expression above, $e\left[t,\theta^{t-1}\right]\approx e\left[t,\theta^{t}\right]$ for slow parameter evolution. The term $\lambda^{t-\tau }$ is a window or weighting function that for λ∈(0,1) assigns more weight to more recent errors [68] (pp. 378–379). The quantity λ is referred to as the “forgetting factor”. The smaller the value of λ, the faster older values of the error (and thus the signal) are forgotten, thus increasing the estimator’s adaptability, i.e., its ability to track the evolution of the dynamics.

The recursive estimation of θ[t] based upon the above criterion is accomplished via the recursive maximum likelihood (RML) method [48,68], thus the models are designated as RML-TAR${\left(na\right)}_{\lambda }$. The estimator update at time *t* based on the previous time instant t−1 is given as:

(12) $$\hat{\theta }\left[t\right]=\hat{\theta }\left[t-1\right]+k\left[t\right]\cdot \hat{e}\left[t|t-1\right]$$

with $\hat{e}\left[t|t-1\right]$ designating the prediction error:

(13) $$\hat{e}\left[t|t-1\right]=y\left[t\right]-\hat{y}\left[t|t-1\right]=y\left[t\right]-\phi^{T}\left[t\right]\cdot \hat{\theta }\left[t-1\right]$$

and k[t] the adaptation gain:

(14) $$k\left[t\right]=\frac{P[t-1]\cdot \phi [t]}{\lambda +\phi^{T}\left[t\right]\cdot P\left[t-1\right]\cdot \phi \left[t\right]}$$

In the Equation (14), P[t] designates the parameter covariance matrix at time *t* that is updated based on the following recursive form:

(15) $$P\left[t\right]=\frac{1}{\lambda }\left[P\left[t-1\right]-\frac{P\left[t-1\right]\cdot \phi \left[t\right]\cdot \phi^{T}\left[t\right]\cdot P\left[t-1\right]}{\lambda +\phi^{T}\left[t\right]\cdot P\left[t-1\right]\cdot \phi \left[t\right]}\right]$$

with ϕ[t] corresponding to the regression matrix of the RML-TAR model, defined as:

$$\phi \left[t\right]=\left[-y[t-1]\;-y[t-2]\;\ldots \;-y[t-na]\right].$$

The method may be initialized with a zero parameter vector $\hat{\theta }\left[0\right]=0$ and unity covariance matrix P[0]=αI, with α designating a “large” positive number. The innovations, i.e., one-step-ahead prediction error, time-varying variance ${\hat{\sigma }}_{e}^{2}\left[t\right]$ may be estimated via a moving window of length 2M+1 centered at each time instant *t* as follows:

(16) $${\hat{\sigma }}_{e}^{2}\left[t\right]=\frac{1}{2M+1}\sum_{\tau =t-M}^{t+M}{\hat{e}}^{2}\left[\tau /\tau -1\right]$$

### 3.2. Model Structure Selection

Model structure selection refers to the selection of the TAR model order na and forgetting factor λ, and is generally based on trial-and-error or successive fitting schemes [48], according to which models corresponding to various candidate structures are estimated, and the one providing the best fitness to the non-stationary signal is selected. The fitness function may be the Gaussian log–likelihood function of each candidate model. The particular model that maximizes it is the most likely to be the actual underlying model responsible for the generation of the measured signal, in the sense that it maximizes the probability of having provided the measured signal values, and is thus selected. A problem with this approach is that the log–likelihood may be monotonically increasing with increasing model orders, and as a result, the over fitting of the measured signal occurs. For this reason, criteria such as the AIC (Akaike information criterion [69]) or the BIC (Bayesian information criterion [70]) are generally used and can be represented as follows:

(17) $$AIC=-2\cdot \ln L(M\left(\theta^{N},{\left(\sigma_{e}^{2}\right)}^{N}\right)|y^{N})+2\cdot d$$

(18) $$BIC=-2\cdot \ln L(M\left(\theta^{N},{\left(\sigma_{e}^{2}\right)}^{N}\right)|y^{N})+\frac{\ln N}{2}\cdot d$$

with L designating the model likelihood, *N* the number of signal samples, and *d* the number of independently estimated model parameters. As it may be observed, both criteria consist of a superposition of the negative log–likelihood function and a term that penalizes the model order, or structural complexity, and thus discourages the model over fitting. Accordingly, the model that minimizes the AIC or the BIC is selected. The Gaussian log–likelihood function of the model structure, $M\left(\theta^{N},{\left(\sigma_{e}^{2}\right)}^{N}\right)\left|y^{N}\right)$, given the signal sample $y^{N}$ is given by:

(19) $$\ln L(M\left(\theta^{N},{\left(\sigma_{e}^{2}\right)}^{N}\right)|y^{N})=\ln f(M\left(y^{N}|\theta^{N},{\left(\sigma_{e}^{2}\right)}^{N}\right))=\ln\prod_{t=1}^{N}f\left(e\left[t\right]|\theta^{N},{\left(\sigma_{e}^{2}\right)}^{N}\right))$$

$$=\sum_{t=1}^{N}\ln\left({\left(2\pi \sigma_{e}^{2}\left[t\right]\right)}^{-\frac{1}{2}}\cdot \exp\left\{-\left(\frac{e^{2}\left[t\right]}{2\sigma_{e}^{2}\left[t\right]}\right)\right\}\right)$$

(20) $$⟹\ln L(M\left(\theta^{N},{\left(\sigma_{e}^{2}\right)}^{N}\right)|y^{N})=-\frac{N}{2}\cdot \ln2\pi -\frac{1}{2}\sum_{t=1}^{N}\left(\ln\sigma_{e}^{2}\left[t\right]+\frac{e^{2}\left[t\right]}{\sigma_{e}^{2}\left[t\right]}\right)$$

As a result, the BIC equation can be written as:

(21) $$BIC=-\frac{N}{2}\cdot \ln2\pi -\frac{1}{2}\sum_{t=1}^{N}\left(\ln\sigma_{e}^{2}\left[t\right]+\frac{e^{2}\left[t\right]}{\sigma_{e}^{2}\left[t\right]}\right)+\frac{\ln N}{2}\cdot d$$

The innovations (one-step-ahead prediction error) variance $\sigma_{e}^{2}\left[t\right]$ may be estimated via Equation (16).

The ratio of the residual sum of squares versus the signal sum of squares (RSS/SSS) may also be used as another fitness criterion for selecting the best model. For determining the best time-varying model, in addition to searching for the best candidate model structure, it is also necessary to search for a suitable forgetting factor for which the minimum RSS/SSS occurs. Again, model order resulting from the least RSS/SSS may provide spurious natural frequencies and damping ratios. As a result, the frozen-time power spectral density for the non-stationary signal may provide additional insight in the selection of the best model structure.

### 3.3. Modal Characteristics

The notion of power spectral density (PSD) for the stationary case has no direct counterpart for the non-stationary case. The frozen-time PSD of a system may be obtained by utilizing a sequence of frozen stationary systems (corresponding to a specific time instant) for representing the non-stationary system [48,59]. By analogy to the stationary case, the power spectral density for each time instant may be expressed based on the estimated TAR model as:

(22) $$S_{F}\left(\omega ,t\right)={\left|\frac{1}{1+\sum_{i=1}^{na}a_{i}\left[t\right]\cdot e^{-j\omega T_{s}i}}\right|}^{2}\cdot \sigma_{e}^{2}\left[t\right]$$

Note that this would be the power spectral density of the response signal if the system were “frozen” at the time instant *t*. As such, the information conveyed is very useful, because it represents the characteristics that the system would have if it became stationary (“frozen”) with a specific configuration (corresponding to the considered time instant *t*).

The normalized TAR model’s “frozen-time” frequency response function can be given by the following equation:

(23) $$H_{F}\left(e^{j\omega T_{s}},t\right)=\frac{1}{1+\sum_{i=1}^{na}a_{i}\left[t\right]\cdot e^{-j\omega T_{s}i}}\cdot \sigma_{e}\left[t\right]$$

The corresponding “frozen” modes with natural frequencies and damping ratios may be given by the following equations [71]:

(24) $$\omega_{ni}\left[t\right]=\frac{\left|\ln\lambda_{i}\left[t\right]\right|}{T_{s}}\;\left(\mathrm{rad}/\mathrm{time}\right),\;\zeta_{i}\left[t\right]=-\cos\left(\arg(\ln\lambda_{i}\left[t\right])\right)$$

with $\lambda_{i}$ designating the *i*-th discrete-time frozen pole.

## 4. Data Generation

### 4.1. The Laboratory Experimental Set-Up

**Figure 2:**
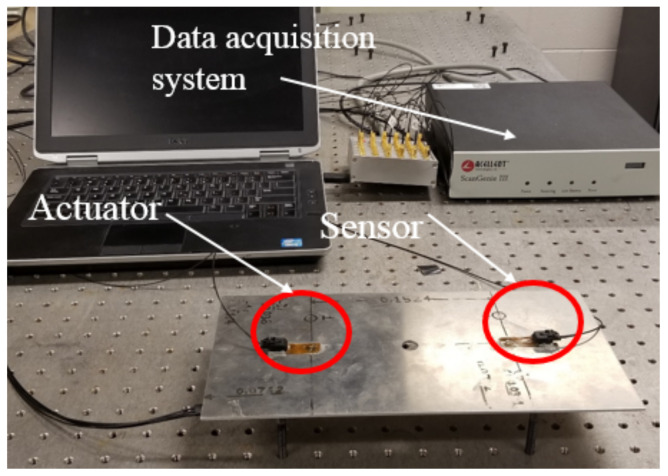
An aluminum plate fitted with the PZT transducers in the pitch-catch mode with the data acquisition system.

The laboratory experimental set-up is shown in Figure 2. It consists of an aluminum plate (Aluminum 6061) with dimensions of 304.8×152.4×2.286 mm (12×6×0.093 in; L×W×H). The plate has a hole in the middle with a diameter of 12 mm (0.5 in). Two single piezoelectric PZT (Lead Zirconate Titanate) transducers were attached to the aluminum coupon by using Hysol EA 9394 adhesive (152.4 mm apart). The PZTs are of type PZT-5A and acquired from Acellent Technologies, Inc. The PZT transducers are 0.2 mm in thickness and 3.175 mm (1/8 inch) in diameter.

The actuation signals used in the PZT transducer were in the form of 5-peak tone bursts (5-cycle Hamming-filtered sine wave) having an amplitude of 90 V peak-to-peak. Various center frequencies of the 5-peak tone burst signal ranging from 200 to 700 kHz were generated in a pitch-catch configuration. With a sampling frequency of 24 MHz, data were collected using a ScanGenie III data acquisition system (Acellent Technologies, Inc., Sunnyvale, california, USA) during 11 days. A thermocouple was mounted on the aluminum plate to record the temperature of the surface during each signal acquisition. A preliminary analysis was conducted, and a center frequency of 250 kHz was chosen for the complete analysis presented in this study based upon the best separation between the first two wave packets. Ambient temperature was maintained in the laboratory that fluctuated based on the day, time, number of people in the lab, and other potential factors. In order to perform the time series modeling, the actual data were down-sampled 12 times to a final sampling frequency $f_{s}$ of 2 MHz and an effective bandwidth of up to 1 MHz as no dynamics are present beyond this frequency. The initial part of the signal (time of flight), which does not contain any dynamics, was discarded for the present analysis. The number of data samples in each collected signal was N=601 (after discarding time of flight portion). All data sets were exported to MATLAB for analysis (MATLAB version R2018a, The MathWorks Inc., Natick, MA, USA).

### 4.2. Finite Element Modeling

**Figure 3:**
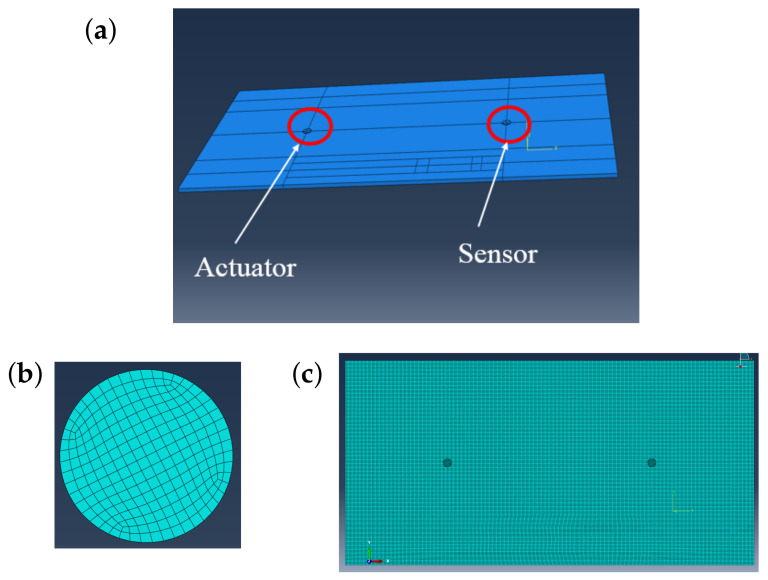
(**a**) FEM model of the aluminum plate with adhesive and PZT sensors; (**b**) meshed view of the PZT sensor; (**c**) the meshed plate with adhesive and PZT sensors.

In order to simulate the effects of temperature on guided wave propagation, a high-fidelity finite element model (FEM) was constructed by using commercial finite element software ABAQUS 2018 (see Figure 3). In the beginning, three separate parts were created namely: an aluminum plate, adhesive, and piezo-electric disk (PZT) following the dimensions given in Table 1. Then, material properties were defined for each part and assigned to the individual sections. The material properties of aluminum, adhesive, and PZT can be found in Table A1. The functional relationship between the temperature and material properties can be found in Appendix A. Once the material properties were assigned to the specific sections, all separate parts were assembled together to form a unified part or model. Then, interactions between different parts were created by defining master and slave surfaces. The interactions between the PZT and adhesive were created by selecting the bottom surface of PZT as the master surface and the top surface of the adhesive as the slave surface. This was implemented for each pair of adhesive and PZT disk.

For the wave propagation simulations, a total time period of 0.0001 s and a time step or increment of $10^{-7}$ s were used. For defining the actuation signal, a 5-peak tone burst with a center frequency of 250 kHz was used. As boundary conditions, the two ends of the aluminum plate were fixed. An electric potential boundary condition was applied to the two end surfaces of the PZT disk. An electric potential of 0 V and 100 V were applied at the bottom and top surfaces of the piezoelectric actuator disk, respectively. This sets up an electric field between the two surfaces of the disk. For the sensor disk, zero voltage was applied only at the bottom surface and the top surface was kept free. 

The electric potential (EPOT) was selected as the response signal from the PZT disk. The output signal can be collected from any single node on the top surface of the PZT sensor; however, signals obtained from different nodes of the surface are different; therefore, it is necessary to obtain the average of all the signals from all the nodes on the top surface of the PZT sensor. This task of averaging can be performed by using equation constraints. Under an equation constraint, a single node is selected as a master node and all other nodes on the surface are constrained with respect to this master node. This forces the electric potential to be the same on all other nodes of the surface that is the same as the master node. It was found that averaging the signals from all the nodes on the surface was equivalent to using the equation constraint where a master node was used to constrain all other nodes on the surface. The coefficients used for the master node and the other nodes were 1 and −1, respectively. The degrees of freedom used for the PZT material was 9.

In order to facilitate meshing, partitioning was performed on all of the three parts, namely, aluminum plate, adhesive material, and PZT disks. For all three parts, a structured hexahedron mesh was used. In order to select the mesh size, a convergence study was performed and a global mesh size of 0.001 was chosen for the aluminum plate. The total number of elements in the plate was 93,330. For the adhesive and PZT material, a global mesh size of 0.0004 was used. The total number of elements in the adhesive and PZT disk were 480. It was ensured that at least 20 elements exist per wavelength. The mesh size can be further reduced, but doing so increases computational cost with no significant increase in accuracy. Linear 3D stress elements (C3D8R) were selected for the aluminum plate and adhesive materials. For the PZT disk, 8-node linear piezo-electric brick elements were used. Implicit dynamics solver has been used for the solution of the guided wave propagation phenomenon instead of the explicit dynamics module, although explicit dynamics solvers are faster. The reason behind using implicit dynamics is that it can handle PZT elements (multi-physics problem) while the explicit dynamics module cannot. With all these specifications, a single simulation requires approximately 6 h to complete. Figure 3 shows the full FEM model of the aluminum plate as well as the mesh of the PZT disk and the plate.

## 5. Results and Discussion

### 5.1. Stochasticity in Guided Wave Propagation

#### 5.1.1. Experimental Guided Wave Signal Analysis

In this section, the statistical analysis of the collected during 11 days of experimental guided wave signals is presented. During these 11 days, the temperature range in the laboratory fluctuated between 21.1 to 25.1 °C. This variation in the ambient temperature of the room was due to the external temperature variation, humidity, different times at which data were collected, varying number of persons working in the laboratory, and potentially other unobservable sources of uncertainty.

**Figure 4:**
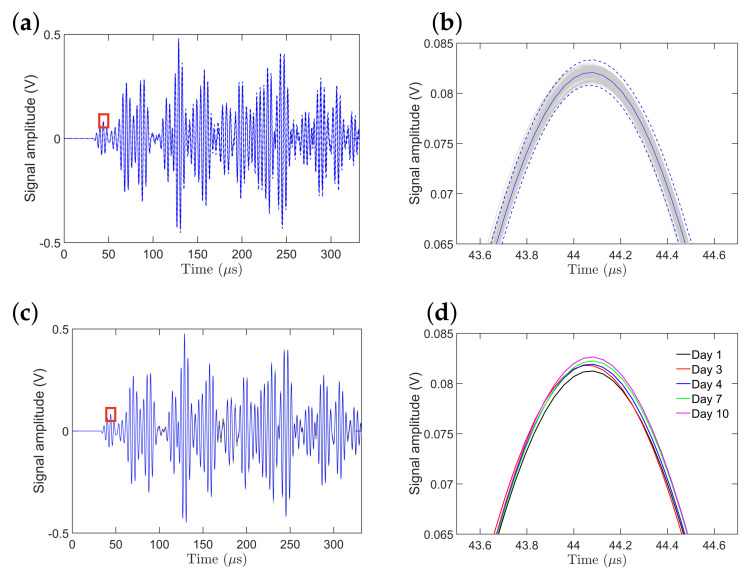
Statistical properties of 440 signals collected during 11 days: (**a**) all the signals with ±3 standard deviations and their mean; (**b**) close–up view of the third peak of the $S_{0}$ mode along with the corresponding ±3 standard deviations; (**c**) signal mean of each day (40 signals per day) for 11 days; (**d**) close–up view of the third peak of the $S_{0}$ mode of the signal mean for selected days.

Figure 4a shows 440 experimental signals, collected during 11 days (during each day 40 data sets were collected at different times), having an initial length of 8000 sample points (not down-sampled) with a signal length of 332 μs. The signal segment from the time instant of 33 μs to 50 μs constitutes the $S_{0}$ mode while the signal segment from 60 μs to 78 μs constitutes the $A_{0}$ mode. After the time instant of 78 μs, the rest of the peaks are considered as coming from the boundary reflections. Figure 4b, that presents a close-up of the third peak of the $S_{0}$ mode, clearly shows the variation between the signals collected during the 11 days. The dotted blue lines represent ±3 standard deviations of the signal. The solid blue line represents the mean of all 440 signals. The amplitude of the third peak of the $S_{0}$ mode, which is the most prominent peak in the $S_{0}$ mode, varies between 0.0808 and 0.0833 V with the mean being 0.0821 V.

**Figure 5:**
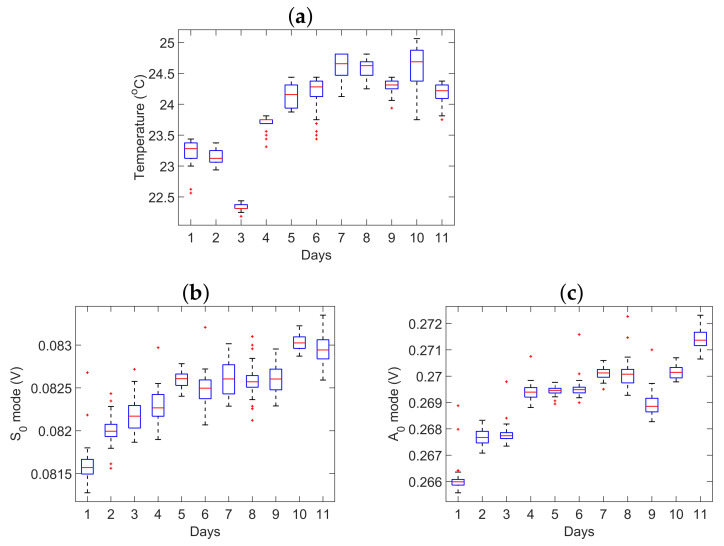
(**a**) Temperature distribution of the 440 signals collected during 11 days (each day 40 signals); (**b**) the Hilbert transform of the highest peak of the $S_{0}$ mode; (**c**) the Hilbert transform of the highest peak of the $A_{0}$ mode.

Figure 4c presents the mean signals (mean of 40 signals) for each day. Figure 4d presents a zoomed-in version of the third peak (highest peak) of the $S_{0}$ mode for a few selected days from Figure 4c. The overall trend of the amplitude of the highest peak of the $S_{0}$ mode can be explained in connection with Figure 5a. Figure 5a shows the temperature distribution for each of the 11 days. The box plot for each day shows the distribution of 40 recorded temperatures, which corresponds to the forty recorded guided wave signals obtained at different times within a single day. The horizontal red line in the boxplots represents the mean value of the temperature distribution. The blue box represents the inter-quartile range, that is, from 25th to 75th percentile values. The two whiskers on both sides represent the range that is 1.5 times the inter-quartile range. The red dots represent the individual outliers or the values that fall outside the 1.5 times the inter-quartile range. From day 1 to 11, an overall upward trend of temperature distribution (from 21.1 to 25.1 °C) can be observed although there are certain exceptions. It can be observed from Figure 4d that the amplitude of the signal gradually increases as the temperature increases in the specified days.

Figure 5b,c represent the maximum value of the Hilbert transform of the amplitude of the highest peak of the $S_{0}$ and $A_{0}$ mode, respectively, collected during 11 days. Here, again, with the increase in temperature, an overall increase in the amplitude of the $S_{0}$ and $A_{0}$ mode can be observed, although a few exceptions exist. This could be due to the aforementioned unobservable factors that may have an effect on the wave propagation and data acquisition process. Indeed, this falls within the objective of this work, to exactly assess and quantify the effects of ambient temperature variations on the propagation of guided waves.

#### 5.1.2. Simulated Guided Wave Signal Analysis

In this section, we present the results based on a series of FEM-based simulations in order to investigate the effect of ambient temperature variation on guided wave propagation and compare the results with the corresponding experimental results. For the performed simulations, it was assumed that the temperature follows a normal distribution; the mean of the distribution was assumed to be 20 °C with a standard deviation of 0.5 °C. Then, 30 random samples were drawn from the distribution and corresponding FEM simulations were performed (it has been shown that at least 28 samples are required to represent a normal distribution [72]). For each drawn temperature point, a separate simulation was conducted by changing 20 material properties (details and equations used are provided in the Appendix A) and the PZT response signals were collected and the statistical analysis was performed to observe the variation in the wave propagation.

**Figure 6:**
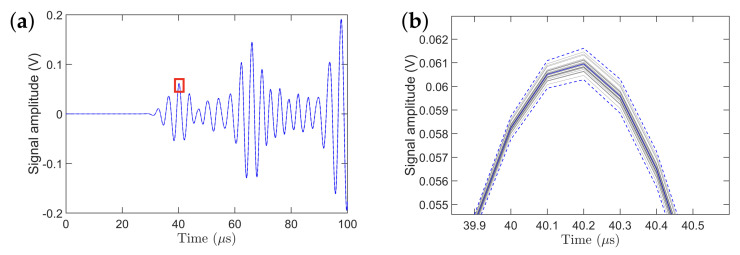
(**a**) Statistical properties of 30 simulated signals; (**b**) close–up view of the third peak of the $S_{0}$ mode. The solid blue line represents the mean of all the signals and the dashed blue lines represent the ±3 standard deviations confidence intervals.

Figure 6a presents the signals obtained from 30 simulations for 30 different temperatures that were drawn from a normal distribution. The signals from the time instant 35 μs to 45 μs constitute the $S_{0}$ mode while the signals from 55 μs to 75 μs constitute the $A_{0}$ mode. After the time instant of 75 μs, the rest of the peaks are considered as coming from the boundary reflections. The amplitude of the third peak of the of the $S_{0}$ mode, which is the most prominent peak in the $S_{0}$ mode, varies between 0.060 and 0.062 V.

Figure 6b presents a close-up of the simulated signals; the dotted blue line on the top and bottom represents the ±3 standard deviations confidence intervals while the solid line represents the mean of all 30 signals. It can be also observed that the standard deviation near the peak of the signal is much higher than the standard deviation of the remaining signal that is offset from the peak. Another important observation from this plot is that the maximum value of the peak occurs at the same time instant, that is, no change in the time of flight (phase) of the wave packet occurs when the standard deviation of the temperature distribution is low. In order to provide a single maximum or minimum value of the signal, a Hilbert transform was performed on the $S_{0}$ and $A_{0}$ modes of all the signals, and their minimum and maximum values were obtained. The results are shown in Table 2. It is observed that the variation in the experimental and simulated signals is on the same order of magnitude (Table 2) with the experimental standard deviation being, as expected, higher than the simulated one. For the simulated signals the variation comes only from the variation in temperature, and no other external sources that may affect the signals are present. As a result, the experimental variation is higher as other sources of uncertainty affecting the wave propagation are present. Note that no efforts were made to update the FEM based on experimental data, as the main objective of this study is to assess the effect of ambient temperature variation on the wave propagation.

### 5.2. Non-Parametric Analysis: Experimental Signal

Before performing the parametric analysis based on TAR models, it is customary to perform the non-parametric analysis as a preliminary straightforward step for an initial analysis of the collected data. Although it may potentially provide a less-accurate estimate, compared to the parametric analysis, of the signal’s frequency and corresponding power content, the results can often be compared with the complex parametric models for validation purposes and to avoid any artifacts that may arise from the suboptimal model order selection and parameter estimation.

Short-time Fourier transform (STFT), also referred to as the “spectrogram”, is the most widely used method for analyzing non-stationary signals. The spectrogram provides information on the non-stationary signal’s time and frequency content. The idea is to break down the non-stationary signal into smaller segments within which the signal may be considered stationary. Then, perform the Fourier transform of the signal’s smaller segments and sum them together to obtain the time and frequency information simultaneously; however, an arbitrarily small time signal cannot be used because it yields a very low frequency resolution. As a result, there is a compromise between the time and frequency resolution of the non-stationary signal [53].

Keeping in mind the above-mentioned compromising facts, the non-parametric analysis of the non-stationary guided wave signals was performed by (spectrogram.m function of MATLAB (MATLAB version R2018a, The MathWorks Inc., Natick, MA, USA)). The analysis is based on 601-sample-long response signals with a sampling frequency of 2 MHz obtained from the piezoelectric sensor mounted on the aluminum plate. A 30-sample-long Hamming data window (frequency resolution Δf=666.66 Hz) with 98% overlap has been used for the spectrogram analysis. The number of FFT points used was 100 times the window size.

**Figure 7:**
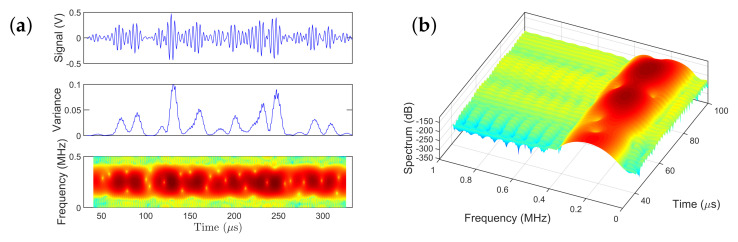
Non–parametric time–dependent power spectral estimates of the non-stationary experimental guided wave signal: (**a**) the guided wave signal (top subplot), time–dependent variance (middle subplot) and the 2D spectrogram (bottom subplot); (**b**) 3D view of the spectrogram.

Figure 7 presents an indicative spectrogram of the non-stationary guided wave signal. The top subplot of Figure 7a presents the guided wave response signal from the PZT transducer. The middle subplot depicts the time-varying variance of the signal. Observe the evident non-stationarity and significant fluctuation of the estimated variance for the selected window size of 20 samples. The bottom subplot shows how the frequency spectrum of the guided wave signal that changes with time during propagation on an aluminum plate (isotropic medium). It can be observed that the power of the guided wave signal is concentrated, as expected, around 250 kHz, which is the center frequency of the actuation signal; however, the power evolves over time due to the fact that the guided waves (Lamb waves) are dispersive in nature. Figure 7b shows the 3D view of the spectrogram.

### 5.3. Parametric RML-TAR Modeling under Ambient Temperature Variation

Once the non-parametric spectrogram analysis was performed, the parametric RML-TAR model identification was subsequently addressed to represent the non-stationary guided wave propagation under ambient temperature variation, extract the time-varying spectral content, and assess the propagated uncertainty onto the estimated model parameters and parameter covariance matrix.

Model selection of RML-TAR involves selecting the appropriate model order na and forgetting factor λ. The RSS/SSS (residuals sum of squares/signal sum of squares) criterion, describing the predictive ability of the model, was employed for the model selection process. AR orders from na=2 to na=22 and forgetting factor values λ∈[0.5,0.999] (with an incremental step of 0.001) were considered to create a pool of candidate models. A total of 10,500 models (500 × 21) were estimated, and among all these models the best model was chosen as the one that minimizes the RSS/SSS. Following this criterion, the best model occurred for na=6 and forgetting factor λ=0.532. This can be compactly represented as RML-TAR${\left(6\right)}_{0.532}$. In addition to the RSS/SSS criterion, the Bayesian information criterion (BIC), which rewards the model’s predictive capability while penalizing model complexity for increasing model order [48], and “frozen-time” natural frequencies were also taken into account. A higher model order may lead to spurious natural frequencies that are unlikely to occur in the system being considered due to the narrowband, deterministic excitation. On the other hand, a lower model order will be unable to capture some of the frequencies that are truly present in the real system.

**Figure 8:**
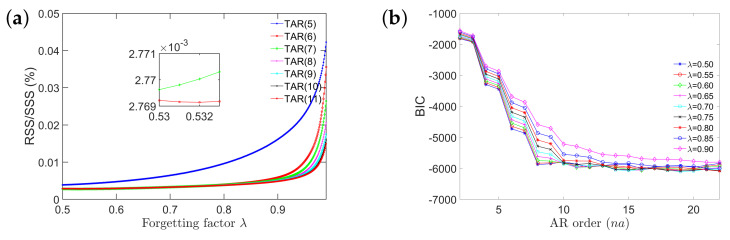
Model order selection: (**a**) the RSS/SSS versus the forgetting factor values for a few selected model orders; (**b**) the BIC values with increasing model order for different forgetting factors.

Figure 8a shows the RSS/SSS versus the forgetting factor plot for a representative data set and a few selected model orders. It can be observed that, for RML-TAR(5), as the value of the forgetting factor increases, the RSS/SSS also increases. For RML-TAR(6) up to RML-TAR(11), the RSS/SSS has a plateau for a forgetting factor value between 0.5 to 0.9, and then sharply increases as it approaches 1. Among all the model orders, RML-TAR(6) with forgetting factor λ=0.532 reaches the minimum RSS/SSS and thus found to be the “best” model. Figure 8b depicts the BIC versus increasing AR order for different forgetting factor values. As the model order increases, the BIC sharply decreases up until AR order na=8 and then reaches a plateau for higher model orders. It can be observed that for a specific model order, the BIC values are higher for the forgetting factors closer to 1. Although selecting a higher model order provides a lower BIC value, it will also provide additional spurious “frozen-time” natural frequencies due to model order overdetermination.

For the estimation of the parameters of the selected model structure, a recursive estimation scheme was used as outlined in Section 3.1. Initially, due to the recursive nature of the parameter estimation method, the estimation accuracy is low as there is a limited number of data points available. In order to minimize these effects, parameter estimation was implemented into three steps, namely: (i) forward pass, (ii) backward pass, and (iii) final forward pass. In the forward pass, initial conditions for the parameters were assumed as zero, and a larger value of the covariance matrix was assumed (P=αI). A larger value of the covariance matrix at the beginning reflects that there is lower confidence in the assumed initial conditions. After several iterations, as more data become available, parameter estimation converges to the true parameter values. In the backward pass, the final values of the signal are used as the initial values and the final estimated parameters and covariance matrix from the forward pass are used as the initial conditions. In the final forward pass, the final estimated parameters and covariance from the backward pass are used as the initial conditions. This helps achieve better parameter estimation at the beginning of time as informed initial conditions are being used in the estimation algorithm. All the results presented in this section are for the final forward pass.

**Figure 9:**
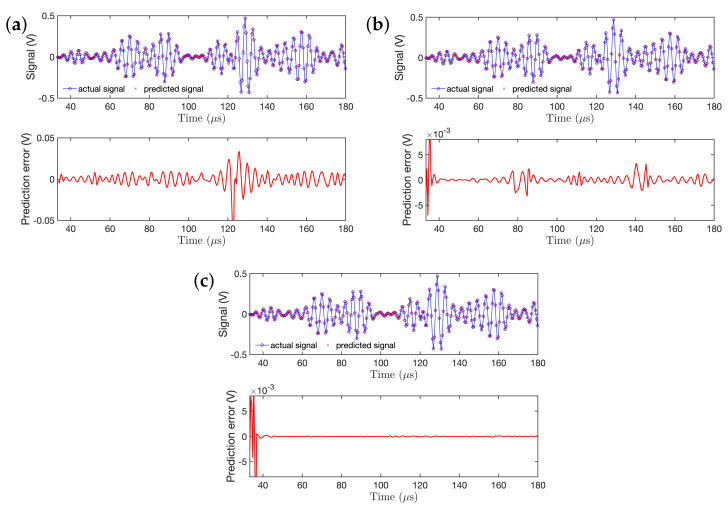
Segment of a non–stationary guided wave signal realization ($S_{0}$, $A_{0}$ mode, and part of the reflected signal) superimposed with RML–TAR–based one–step–ahead prediction, and the corresponding residuals: (**a**) RML–TAR${\left(2\right)}_{0.532}$; (**b**) RML–TAR${\left(4\right)}_{0.532}$; (**c**)RML–TAR${\left(6\right)}_{0.532}$.

Upon consideration of these factors, the best model was selected for the subsequent analysis. Figure 9 presents the RML-TAR based one-step-ahead predictions of a short segment of the guided wave signal ($S_{0}$, $A_{0}$ mode, and part of reflection), and their corresponding prediction errors. It can be observed from Figure 9a that the prediction error for RML-TAR${\left(2\right)}_{0.532}$ is relatively higher (on the order of $10^{-4}$) while the model does not pass the residual validation test (for details see [48]). That is, RML-TAR${\left(2\right)}_{0.532}$ is not fully capturing the system dynamics. With the increase in model order (Figure 9b) and a RML-TAR${\left(4\right)}_{0.532}$ model, the prediction error reduces to the order of $10^{-5}$. For the RML-TAR${\left(6\right)}_{0.532}$ model (Figure 9c), the prediction error reduces to the order of $10^{-6}$ and the model is properly validated.

**Figure 10:**
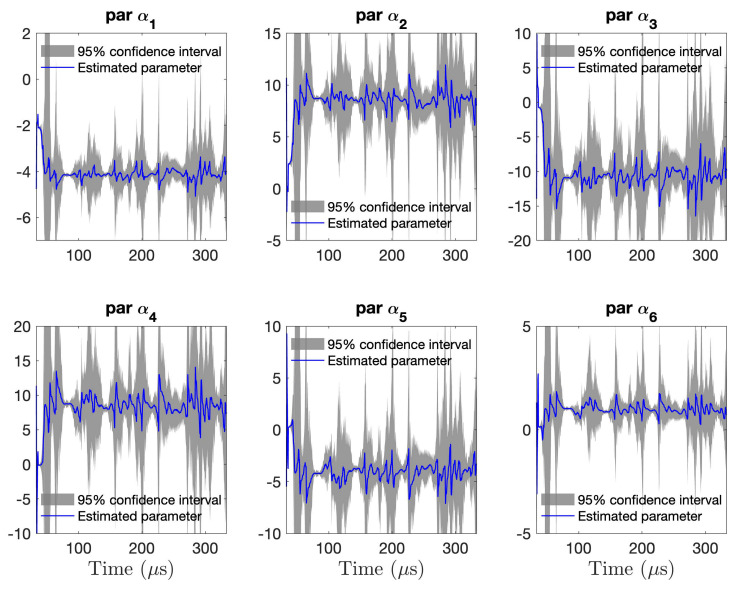
Confidence intervals of the estimated time–varying model parameters for a single non–stationary guided wave signal realization: The blue lines represent the estimated time–varying RML–TAR${\left(6\right)}_{0.532}$ parameters while the shaded areas represent the corresponding 95% confidence intervals based on the time–varying parameter covariance matrix.

Based on the above, the RML-TAR${\left(6\right)}_{0.532}$ model structure was selected for the subsequent analysis. During the RML estimation process, the time-varying covariance of the model parameters is also estimated. Figure 10 depicts the six estimated parameters of the RML-TAR${\left(6\right)}_{0.532}$ model with their corresponding confidence intervals obtained via the estimated parameter covariance matrix. The blue solid lines represent the estimated model parameters and the grey shaded regions represent their corresponding 95% confidence interval. Unlike Figure 9, where the signal is shown up to 180 μs, the model parameters are shown until the end of the recorded signal (332 μs). Observe the time-varying confidence intervals leading to segments of increased estimation uncertainty.

**Figure 11:**
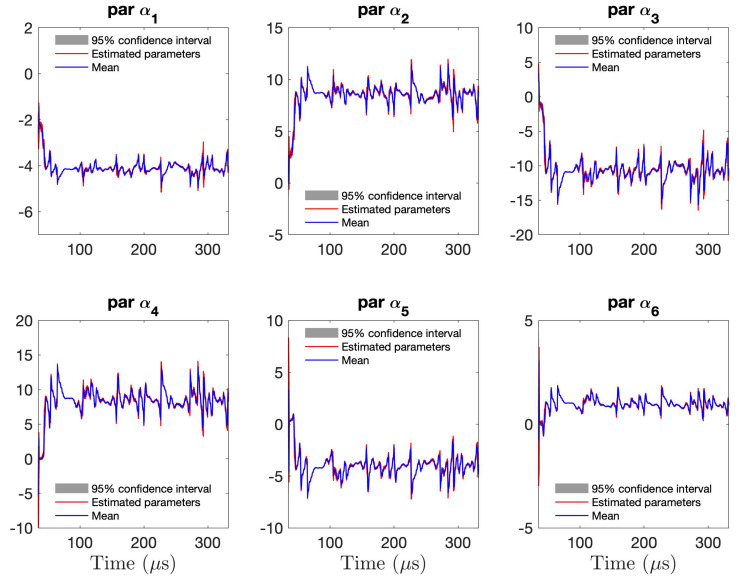
Time–varying parameters estimated by RML–TAR${\left(6\right)}_{0.532}$ models for all 440 experimental signals: time–varying mean of all 440 parameters (blue) along with all estimated parameters (red) and ±2 standard deviations (95% confidence intervals).

**Figure 12:**
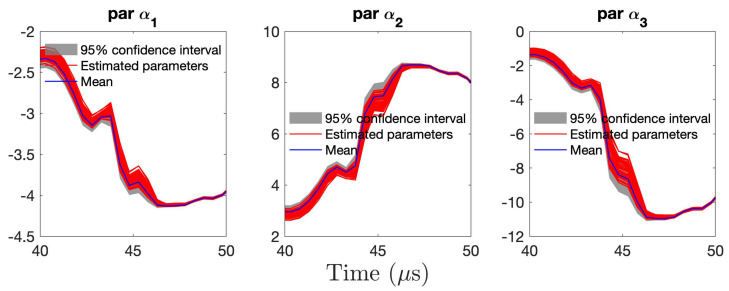
Close–up version of Figure 11 with the first three time–varying parameters estimated by RML–TAR${\left(6\right)}_{0.532}$ for all 440 experimental signals: time–varying mean parameter (blue) along with all 440 estimated parameters (red) and ±2 standard deviations.

Figure 11 presents the RML-TAR${\left(6\right)}_{0.532}$ estimated parameters for all 440 guided wave signals collected from the piezoelectric sensors during the 11 days. For clarity, a zoomed-in version of the first three parameters is shown in Figure 12. The red lines correspond to the estimated time-varying parameters for all 440 guided wave signals obtained during the 11 days of the experiment. The solid blue lines correspond to the time-varying mean of the 440 estimated parameters. The grey shaded region represents the 95% confidence intervals of the 440 estimated parameters. It can be observed that the variation of the model parameters is quite small for the experimental temperature variation. Based on these figures, it can also be observed that the model estimation uncertainty, as represented by the time-varying covariance matrix, is higher than the corresponding experimental uncertainty reflected by estimation of models corresponding to each of the 440 collected data sets.

**Figure 13:**
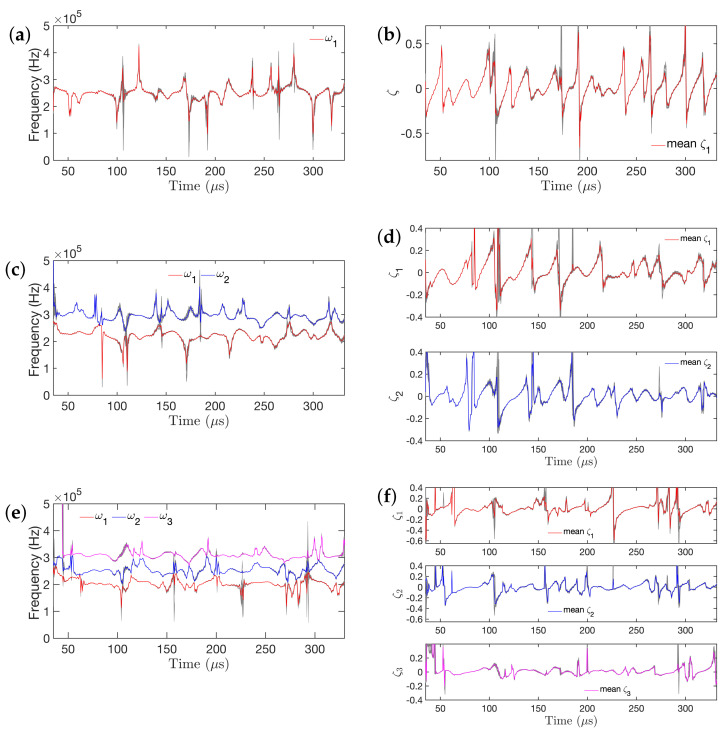
“Frozen–time” natural frequencies and damping ratios of the estimated models for the 440 experimental signals collected during 11 days under ambient temperature variation: (**a**,**b**) RML–TAR${\left(2\right)}_{0.532}$; (**c**,**d**) RML–TAR${\left(4\right)}_{0.532}$; (**e**,**f**) RML–TAR${\left(6\right)}_{0.532}$ model. The red, blue, and magenta lines represent the mean “frozen–time” natural frequencies and damping ratios, and the grey lines represent other 440 “frozen–time” natural frequencies and damping ratios.

Figure 13 depicts a comparison of the “frozen-time” natural frequencies and corresponding damping ratios obtained from the RML-TAR${\left(2\right)}_{0.532}$, RML-TAR${\left(4\right)}_{0.532}$, and RML-TAR${\left(6\right)}_{0.532}$ models. The grey lines represent the “frozen-time” natural frequencies based on the estimated models for all 440 experimental guided wave signals, and the red, blue, and magenta lines represent the mean time-varying natural frequencies and damping ratios. It should be noted that a 5-peak tone burst signal with a center frequency of 250 kHz was used as the excitation signal. As a result, the spectrum of the output signal is expected to be concentrated around 250 kHz. Figure 13a,b show the natural frequencies and damping ratios, respectively, extracted from the RML-TAR${\left(2\right)}_{0.532}$ models. In this case, as na=2, only one frequency and damping ratio is present. Figure 13c,d show the natural frequencies and damping ratios, respectively, obtained from the estimated RML-TAR${\left(4\right)}_{0.532}$ models. In this case, two natural frequencies and damping ratios are present that exhibit a small experimental variation. Similarly, Figure 13e,f show the natural frequencies and damping ratios, respectively, as obtained from the RML-TAR${\left(6\right)}_{0.532}$ models of the 440 data sets. In this case, the first natural frequency occurs at 200 kHz, the second one occurs at 250 kHz, and the third around 300 kHz. Again, the “frozen-time” natural frequencies and damping ratios do not remain constant but also evolve over time. It can be observed that the damping ratios become positive and negative in a periodic fashion. The presence of this periodic positive and negative damping may indicate the presence of periodic stability and instability in the system that may arise from the nature of the wave propagation and the energy exchange between the wave and its reflections.

**Figure 14:**
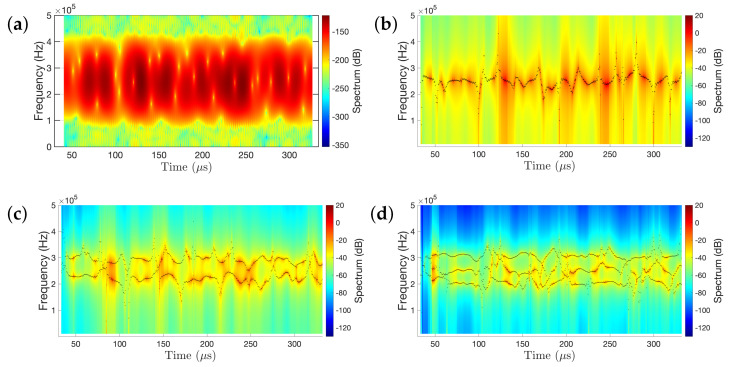
Non–parametric spectrogram and parametric “frozen–time” frequency response function comparison: (**a**) indicative spectrogram; (**b**–**d**) the top view of the 3D frequency response function (FRF) estimated by (**b**) RML–TAR${\left(2\right)}_{0.532}$; (**c**) RML–TAR${\left(4\right)}_{0.532}$; (**d**) RML–TAR${\left(6\right)}_{0.532}$ along with their corresponding natural frequencies.

Figure 14 presents the comparison between the non-parametric spectrogram and the “frozen-time” parametric frequency response functions (FRFs) obtained from different RML-TAR models. From Figure 14a, it can be observed that the frequency spectrum is concentrated between 200 and 300 kHz and its amplitude varies over time. The parametric FRFs, estimated by RML-TAR${\left(2\right)}_{0.532}$ (Figure 14b), RML-TAR${\left(4\right)}_{0.532}$ (Figure 14c), and RML-TAR${\left(6\right)}_{0.532}$ (Figure 14d), RML-TAR${\left(6\right)}_{0.532}$-based model provide a more detailed representation compared to the non-parametric spectrogram with RML-TAR${\left(6\right)}_{0.532}$ being able to provide the best representation of the narrowband dynamics of the wave propagation signal.

**Figure 15:**
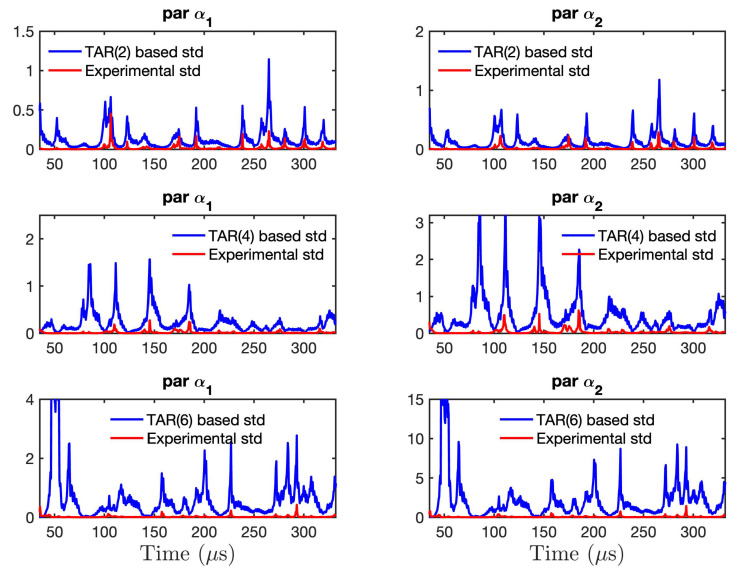
Comparison between RML–TAR model-based parameter standard deviation and experimental standard deviation derived from the model parameters of 440 RML–TAR models. Observe that the experimental standard deviation is lower than the model–based (TAR–based) standard deviation, that is, model–based uncertainty encompasses the experimental uncertainty.

Figure 15 shows the comparison between the model-based (TAR-based) standard deviation, obtained from the estimated time-varying model parameter covariance matrix, and the experimental standard deviation for the first two model parameters derived from the RML-TAR${\left(2\right)}_{0.532}$, RML-TAR${\left(4\right)}_{0.532}$, and RML-TAR${\left(6\right)}_{0.532}$ models. It can be observed that the model-based covariance matrix, due to estimation uncertainty, and the corresponding standard deviation is over-estimating the experimental uncertainty, and as the model order increases, the standard deviation also increases. As a result, based on the selected RML-TAR model structure, it is possible to model and capture the uncertainty arising from small temperature variations by using a single experimental realization; an RML-TAR model, obtained from a single experiment out of the 440 available data sets, is able to capture the experimental uncertainty during the 11 days and, in fact, overestimates it. This is due to the inherent capability of stochastic time series models to represent estimation and experimental uncertainties. In addition, the use of deterministic actuation signals enhances this ability.

## 6. Concluding Remarks

In this study, an investigation on the variability of the guided wave propagation due to ambient temperature variation based on the stochastic modeling of guided wave propagation using the principles of system identification was performed. A series of laboratory experiments were conducted to record the guided wave signals in the ambient with the corresponding temperature of the individual guided wave signal. In addition, a high-fidelity finite element model was established to model the variability in guided wave propagation signal due to small temperature variation. Monte Carlo simulations were performed assuming that temperature variation follows a normal distribution with a specific mean and variance. The effect of this temperature variation on the input parameters such as Young modulus, Poisson ratio, density, etc., was propagated to the response guided wave signal. It was found that the standard deviation of the experimental signal and the simulated signal is on the same order of magnitude. As a result, it is inferred that the variation in guided wave propagation in the ambient mostly stems from the temperature variation.

Next, non-stationary time-varying time series models, based on the RML-TAR model structure, were employed to represent the non-stationary guided wave signals. RML-TAR models exhibit unstructured parameter evolution as no restriction is imposed on the evolution of these stochastic time-varying parameters. The capability of the RML-TAR models for representing the underlying dynamics and response characteristics of the structure, based on a single non-stationary guided wave signal response was also demonstrated. Different RML-TAR models, i.e., RML-TAR${\left(2\right)}_{0.532}$, RML-TAR${\left(4\right)}_{0.532}$, and RML-TAR${\left(6\right)}_{0.532}$ were identified and compared. The RML-TAR${\left(6\right)}_{0.532}$-based “frozen-time” FRF and modal parameter estimates are both in very good agreement with their non-parametric “frozen-configuration” counterparts, that is, with the spectrogram. As this work is mainly focused on parametric modeling of ultrasonic guided wave propagation under ambient temperature variation, the spectrogram has been used as a means of non-parametric time–frequency analysis. Other types of time–frequency analysis such as Wigner–Ville distribution, XWT and LSCWA can also be considered. It has been shown that the RML-TAR${\left(6\right)}_{0.532}$- based “frozen-time” FRF is able to capture the modal information. Most importantly, the RML-TAR-based parameter confidence bounds, estimated from a single guided wave propagation signal, overestimates the corresponding experimental confidence bounds and can be used as a means to model and capture experimental variation.

It should be stressed here that, in this study, for the generation of the guided wave signal, a 5-peak tone-burst (narrowband) signal with an excitation frequency of 250 kHz was used as the actuation. This actuation results in symmetric ($S_{0}$) and anti-symmetric ($A_{0}$) modes, which enable the use of traditional SHM methods and established damage diagnostic tools; however, from a system identification point of view, a white noise actuation may perform better for the identification of the system as the broadband excitation provides a much richer dynamic content that is also required for the effective identification based on the persistence of excitation concept [68]. In a preliminary study by the authors, it was shown that the Gaussian random white noise actuation implemented on a PZT actuator mounted on an aluminum plate generates a rich stochastic signal with evident structure-sensor-adhesive dynamics and involves a wave mode conversion [42]. In that case, more elaborate stochastic signal processing and modeling tools are required and an appropriate damage diagnostic scheme utilizing such stochastic techniques is necessary. This is the topic of ongoing work and will be presented in future studies by the authors. The RML-TAR modeling approach presented herein will pave the way for the postulation of a thorough SHM framework that accounts for uncertainty due to small changes in ambient temperature with the ultimate aim of integrating this analysis with active sensing SHM technologies in order to increase their robustness and subsequent reliability.

## Figures and Tables

**Table 1:** Dimensions of aluminum plate, PZT sensors, and adhesive.

Object	Dimension
Thickness of aluminum plate	2.286 mm
Thickness of PZT sensor	0.25 mm
Diameter of PZT sensor	6.35 mm
Thickness of adhesive	0.05 mm
Length of aluminum plate	304.8 mm
Width of aluminum plate	152.4 mm

**Table 2:** Statistics of the $S_{0}$ and $A_{0}$ modes due to ambient temperature variation.

	$S_{0}$ (Simulation)	$A_{0}$ (Simulation)	$S_{0}$ (Experiment)	$A_{0}$ (Experiment)
Maximum	0.0618	0.1464	0.0833	0.2722
Minimum	0.0609	0.1451	0.0808	0.2634
Mean	0.0613	0.1457	0.0821	0.2678
Standard deviation	$2.088\times 10^{-4}$	$2.796\times 10^{-4}$	$8.33\times 10^{-4}$	$2.93\times 10^{-3}$

The unit is V.

## Data Availability

Not applicable.

## Abbreviations

The following abbreviations are used in this manuscript:

ACF	Auto-correlation function
AIC	Akaike information criteria
AR	Autoregressive
ARMA	Autoregressive moving average
ARMAX	Autoregressive moving average with exogenous excitation
BIC	Bayesian information criterion
CCF	Cross-correlation function
EOC	Environmental and operational conditions
EPOT	Electric potential
FEM	Finite element model
FRF	Frequency response function
iid	identically independently distributed
MA	Moving average
MMSE	Minimum mean square error
LSCWA	Least squares cross-wavelet analysis
LSWA	Least squares wavelet analysis
OLS	Ordinary least squares
PEM	Prediction error method
PSD	Power spectral density
PZT	Lead zirconate titanate
RML	Recursive maximum likelihood
RSS	Residual sum of squares
SEM	Spectral element method
SHM	Structural health monitoring
SPP	Samples per parameter
SSS	Signal sum of squares
STFT	Short term Fourier transform
TAR	Time-varying autoregressive
TARMA	Time-varying autoregressive moving average
ToF	Time of Flight
WLS	Weighted least squares
XWT	Cross-wavelet transform

## Appendix A. Material Properties and Equations

The established functional relationships are outlined in the following.

Properties of piezoelectric materials:

$$\frac{\partial \rho }{\partial T}=7751.80-7.26\times 10^{-2}T$$

$$E_{11}=E_{22}=60.45+2.09\times 10^{-2}T$$

$$E_{33}=52.95+9.8\times 10^{-3}T$$

$$\nu_{13}=\nu_{23}=0.43+3\times 10^{-4}T-3\times 10^{-6}T^{2}-1\times 10^{-9}T^{3}$$

$$\nu_{12}=0.35+2\times 10^{-4}T-8\times 10^{-7}T^{2}+2\times 10^{-9}T^{3}$$

$$d_{31}=d_{32}=170.78-7.1\times 10^{-3}T+6\times 10^{-4}T^{2}+2\times 10^{-16}T^{3}$$

$$d_{33}=369.12+1.49\times 10^{-1}T+1.9\times 10^{-3}T^{2}-4\times 10^{-9}T^{3}$$

$$d_{15}=d_{24}=556+4.9\times 10^{-2}T+2\times 10^{-6}T^{2}-2\times 10^{-9}T^{3}$$

$$\varepsilon_{11}=\varepsilon_{22}=14.9\times 10^{-9}+1.42\times 10^{-11}T+9.74\times 10^{-14}T^{2}+4.43\times 10^{-17}T^{3}$$

$$\varepsilon_{33}=14.60\times 10^{-9}+1.47\times 10^{-11}T+1.18\times 10^{-13}T^{2}-5.31\times 10^{-18}T^{3}$$

Properties of aluminum:

$$E_{Al}=69.62-2.63\times 10^{-2}T$$

$$\frac{\partial \rho }{\partial x}=2794.60-1.84\times 10^{-1}T$$

$$\nu_{Al}=0.32+3\times 10^{-4}T$$

Properties of the adhesive:

$$E_{adh}=3.2-0.065T+1.18\times 10^{-3}T^{2}-7.72\times 10^{-6}T^{3}$$

$$G_{adh}=1+0.001T-4\times 10^{-5}T^{2}$$

**Table A1:** Nominal material property values at 25 °C.

Materials	Property Name	Values
Piezo-electric: PZT-5A	Density (ρ)	7750 kg/m${}^{3}$
	Young’s modulus (GPa)	$E_{11}=E_{22}=60.97$
$E_{33}=53.19$		
	Poisson ratio	$\nu_{13}=\nu_{23}=0.4402,\nu_{12}=0.35$
	Piezo-elctric charge constant (m/V)	$d_{31}=d_{32}=171\times 10^{-12},d_{33}=374\times 10^{-12},$ $d_{15}=d_{24}=558\times 10^{-12}$
	Dielectric constant	$\epsilon_{11}=\epsilon_{22}=15.32\times 10^{-9},\epsilon_{33}=15\times 10^{-9}$
Aluminum	Density (ρ) (kg/m${}^{3}$)	2700
	Young’s modulus (*E* GPa)	68.9
	Poisson ratio	0.33
Adhesive	Density (ρ) (kg/m${}^{3}$)	1100
	Young’s modulus (*E* GPa)	2.19
	Poisson ratio	0.30
